# Identification and characterization of new *Muscodor* endophytes from gramineous plants in Xishuangbanna, China

**DOI:** 10.1002/mbo3.666

**Published:** 2018-06-21

**Authors:** Li‐Juan Mao, Jia‐Jie Chen, Chen‐Yang Xia, Xiao‐Xiao Feng, De‐Dong Kong, Zhen‐Yu Qi, Feng Liu, Dian Chen, Fu‐Cheng Lin, Chu‐Long Zhang

**Affiliations:** ^1^ State Key Laboratory for Rice Biology Institute of Biotechnology Zhejiang University Hangzhou China; ^2^ Analysis Center of Agrobiology and Environmental Science Zhejiang University Hangzhou China; ^3^ Agricultural Experiment Station Zhejiang University Hangzhou China; ^4^ Naban River Watershed National Nature Reserve Jinghong China

**Keywords:** antifungal activity, internal transcribed spacer of ribosomal DNA, *Muscodor*, volatile organic compounds

## Abstract

The endophytic fungi *Muscodor* spp. produce volatile organic compounds (VOCs) which can inhibit and even kill pathogenic fungi, bacteria, and nematodes. Nine endophytic fungal strains, isolated from the shoots of gramineous plants including *Arthraxon hispidus*,* Eleusine indica*,* Oplismenus undulatifolius*, and *Oryza granulata*, were identified as *Muscodor* through phylogenetic analysis of the internal transcribed spacer. Through an SPSS K‐means cluster analysis, the nine *Muscodor* strains were divided into four groups based on the antifungal activities of the VOCs produced by these fungi determined by a two‐section confrontation test. The first group contains the strains Y‐L‐54, W‐S‐41, Y‐S‐35, W‐T‐27, and Y‐L‐56, which showed the strongest activity. The second and third groups contain W‐S‐35 and Y‐L‐43, which showed stronger and moderate activity, respectively. The fourth group contains W‐S‐38 and N‐L‐7, which were the weakest in inhibiting the tested pathogens. Thirty‐five compounds and the relative amounts of VOCs were determined by SPME‐GC‐MS and comparison with the NIST14 mass spectrometry database and Agilent MassHunter qualitative and quantitative analyses. These 35 compounds were classified into two different categories: (a) the product of fatty acid degradation, and (b) the intermediate and final metabolite of the metabolic pathway with the precursor of mevalonic acid. SPSS clustering analysis showed that the chemical components of VOCs might be correlated with their bioactivity rather than their phylogenetic assignment and some of the identified compounds might be responsible for antifungal activity. In conclusion, new *Muscodor* endophytes were recorded in tropical gramineous plants and a number of strains showed remarkable bioactive properties. Therefore, they have important potential applications in the fields of plant disease control.

## 1 INTRODUCTION

Xylariaceous fungi are the dominant group of endophytic fungi. Strobel et al. found that xylariaceous fungal isolate 620 can produce volatile organic compounds (VOCs) with strong antimicrobial activity, has intertwined rope‐like mycelia, and does not produce spores. Therefore, the genus *Muscodor* was established based on its mycelia type and its production of VOCs (Strobel, Dirkse, Sears, & Markworth, 2001; Worapong et al., 2001). *Muscodor* has a significant inhibitory effect or even lethal effect on a variety of pathogens (fungi, bacteria, and nematodes), therefore *Muscodor* has important potential applications in the fields of agriculture and environmental protection (Strobel, 2006). *Muscodor albus* strain QST 20799 and three end products, andante, arabesque, and glissade, were proposed for postharvest fruit, seed, and soil‐borne diseases control. This was approved by the United States Environmental Protection Agency in 2005.

Over the course of exploration of endophytic fungal resources, a total of 21 *Muscodor* species were recorded to date: *M. albus* (Worapong et al., 2001), *M. roseus* (Worapong, Strobel, Daisy, & Castillo, 2002), *M. vitigenus* (Daisy et al., 2002), *M. crispans* (Mitchel, Strobel, Hess, Vargas, & Ezra, 2008), *M. yucatanensis* (Gonzalez et al., 2009), *M. fengyangensis* (Zhang et al., 2010), *M. cinnamomi* (Suwannarach, Bussaban, Hyde, & Lumyong, 2010), *M. sutura* (Kudalkar, Strobel, Riyaz‐Ul‐Hassan, Geary, & Sears, 2012), *M. equiseti*,* M. musae*,* M. oryzae*, and *M. suthepensis* (Suwannaracha et al., 2013), *M. kashayum* (Meshram, Kapoor, & Saxena, 2013), *M. darjeelingensis* (Saxena, Meshram, & Kapoor, 2014), *M. strobelii* (Meshram, Saxena, & Kapoor, 2014), *M. heveae* (Siri‐udom, Suwannarach, & Lumyong, 2015), *M. indicus* and *M. ghoomensis* (Meshram, Gupta, & Saxena, 2015a), *M. tigerii* (Meshram, Gupta, & Saxena, 2015b), *M. coffeanum* (Hongsanan et al., 2015), and *M. camphorae* (Meshram, Kapoor, Chopra, & Saxena, 2017). In a previous study of endophytic fungi from *Oryza granulata* collected from Xishuangbanna, southwest China, which is an area known to contain rich fungal biodiversity, we isolated two *Muscodor* strains (Yuan et al., 2011). From 2015 to 2016, further exploration of the endophytic fungi from gramineous plants, including *Arthraxon hispidus*,* Eleusine indica*,* Oplismenus undulatifolius*, and *Oryza granulata*, from Xishuangbanna, we found that nine endophytic fungal strains can produce VOCs with antifungal activity, has intertwined rope‐like mycelia, and does not produce spores. These phenotypic characteristics suggested that these strains could be belonging to the genus *Muscodor*. Here, we report the identification, antifungal activity, and analysis of VOCs of these nine strains.

## 2 MATERIALS AND METHODS

### 2.1 Endophytic fungi isolation and storage

The gramineous plants were sampled during 2015 and 2016 from Xishuangbanna in Yunnan province of China (E 100°32′–100°44′, N 22°04′–22°17′). The samples were placed in zip‐lock plastic bags, stored in a box with ice and transported to laboratory within 48 hr after sampling. Healthy plant tissues were rinsed with tap water, then immersed in 75% ethanol for 3–5 min and then in 1% sodium hypochlorite for 8–10 min, and finally rinsed thrice with sterile distilled water. The surface disinfected tissues were cut into segments of about 5 mm length and the segments were placed on MEA plate (2% malt extract agar with 50 mg/L chloramphenicol). The plates were then incubated at 25°C in darkness. Fungal hyphae emerging from the segments were transferred to new potato dextrose agar (PDA) plates for purification. The purified cultures were grown on PDA slant, then covered with sterile liquid paraffin, and deposited in Fungal Biology Laboratory of Zhejiang University.

### 2.2 Phylogenetic analysis based on the internal transcribed spacer of ribosomal DNA sequence

The internal transcribed spacer (ITS) was PCR‐amplified using the universal primers, ITS‐1 (5′‐TCCTCCGCTTATTGATATGC‐3′) and ITS‐4 (5′‐TCCTCCGCTTATTGATATGC‐3′) (White, Bruns, Lee, & Taylor, 1990). The PCR amplification products were purified and sequenced bidirectionally on an ABI 3730 sequencer (Applied Bio‐Systems) according to Zhang et al. (2010). The ITS sequences of the type strains of the known *Muscodor* species were retrieved from the National Center for Biotechnology Information's (NCBI) GenBank. ITS sequences obtained in this study were aligned with sequences of known *Muscodor* species using clustal X 2.1 (Larkin et al., 2007) and manually corrected using GeneDoc (Nicholas & Nicholas, 1997). Sequences obtained in this study were deposited in NCBI GenBank with Accession Number MG309792–MG309800.

Phylogenetic analyses were carried out using maximum parsimony (MP) approach with PAUP* v. 4.0b10 (Swofford, 2002) and Bayesian analyses (BI) approach with MrBayes v3.2.6 (Ronquist et al., 2012). jModelTest was used to compare the likelihood of different nested models of DNA substitution and select the best‐fit model for the dataset. Likelihood settings from the best‐fit model (TrN + G) were selected by AIC using jModeltest. The branches were indicated with MP bootstrap proportion (MPBP) and BI posterior probability (BIPP).

### 2.3 Determination of the colony characteristics and optimal growth temperature

The tested *Muscodor* strains (Table 1) were incubated on PDA medium at 25°C for activation. The mycelial disks (5‐mm diameter) were excised from the edge of the *Muscodor* colony, one disk was inoculated on PDA in the center of the Petri dish (9‐cm diameter). The inoculated Petri dishes were kept in darkness, with three kept at each of the following temperatures: 15, 20, 23, 25, 28, 30, 35, and 40°C, respectively. After incubation for 15 days, the colony characteristics were observed and the diameters were recorded.

**Table 1:** The *Muscodor* endophytes included in this study

Strain	Species	Host	Tissue	ITS GenBank accession number
Y‐L‐54	*Muscodor* sp.1	*Oryza granulata*	Leaf	MG309797
W‐S‐41	*Muscodor* sp.1	*Arthraxon hispidus*	Sheath	MG309800
W‐T‐27	*Muscodor* sp.2	*Oplismenus undulatifolius*	Stalk	MG309795
Y‐S‐35	*Muscodor* sp.2	*Oryza granulata*	Sheath	MG309796
W‐S‐38	*Muscodor* sp.3	*Oplismenus undulatifolius*	Sheath	MG309799
N‐L‐7	*Muscodor* sp.4	*Eleusine indica*	Leaf	MG309792
Y‐L‐43	*Muscodor* sp.4	*Oryza granulata*	Leaf	MG309793
W‐S‐35	*Muscodor* sp.4	*Oplismenus undulatifolius*	Sheath	MG309794
Y‐L‐56	*Muscodor* sp.5	*Oryza granulata*	Leaf	MG309798

*Note*

ITS: internal transcribed spacer.

### 2.4 Assessment of the antifungal activity of VOCs

To measure antifungal activity, five plant pathogenic fungi, *Botrytis cinerea* strain ZJUP10, *Fusarium oxysporum* strain ZJUP28, *Penicillum digitatum* strain Pd01, *Pytium ultimum* strain ZJUP22, and *Pestalotia diospyr* strain ZJUP21 were used. The tested pathogens were collected and preserved in the State Key Laboratory for Rice Biology at Zhejiang University, China.

The antifungal activity of the VOCs produced by the tested *Muscodor* strains was determined by confrontation culture in a Petri dish with two‐sections. A mycelial disk with a diameter of 5 mm was taken from the edge of the *Muscodor* culture, and then incubated on PDA in one section of the Petri dish at 25°C for 4 days in darkness, and a pathogenic fungal mycelia disk of same size was placed in the other section. The Petri dishes were wrapped with two layers of Parafilm and incubated at 25°C for 4 days in darkness. In parallel, the tested pathogens were grown in the absence of *Muscodor*, and when their colonies reached the edge of the Petri dish, the diameters of the corresponding pathogenic fungal colonies in the confrontation treatment were measured. To evaluate the viability of the tested pathogens, the abovementioned pathogen colonies were transferred onto new PDA plates and incubated at 25°C to test their viability.

### 2.5 Determination of the components and the relative amounts of VOCs

*Muscodor* strains were incubated on PDA in Petri dishes with two sections as described above. The PDA plate without *Muscodor* was considered a control. The VOCs emitted by *Muscodor* were extracted with an SPME syringe (SUPELCO) 50/30 mm divinylbenzene/carboxen/polydimethylsiloxane on StableFlex fiber. VOCs were analyzed by GC‐MS (Agilent 6890N/5975B) following procedures described in Zhang et al. (2010). The data were processed using the Agilent MassHunter workstation (Agilent). Chromatographic peaks were recognized by Agilent MassHunter qualitative analysis B.07.00, and the mass spectrogram was obtained. Then, the compounds were identified by a search performed in the NIST14 database. A quantitative analysis of VOCs produced by *Muscodor* strains was performed through Agilent MassHunter quantitative analysis b.07.01.

### 2.6 Data statistical analysis

Statistical analyses were conducted using IBM SPSS 22.0. The antifungal activities of nine *Muscodor* strains against five plant pathogenic fungi were analyzed using an SPSS K‐means cluster analysis. The components and the relative amounts of VOCs produced by these *Muscodor* strains were analyzed through SPSS clustering analysis with the nearest neighbor element and Pearson correlation, with a range from 0 to 1.

## 3 RESULTS

### 3.1 Phylogenetic analysis of the endophytic fungal strains

**Figure 1:**
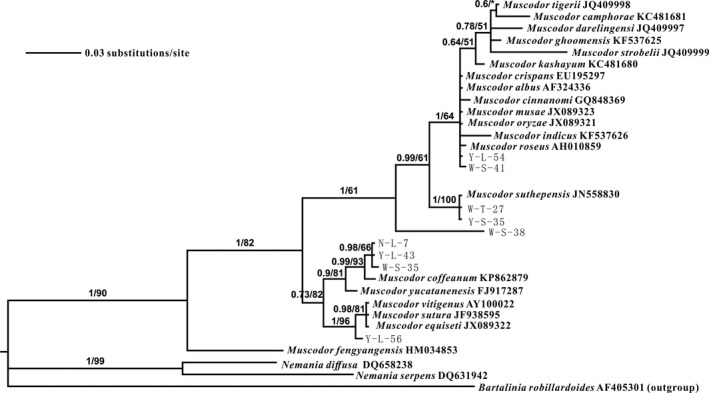
Phylogenetic tree of *Muscodor* spp. based on internal transcribed space sequences. The branches are indicated with maximum parsimony bootstrap proportion and Bayesian analyses posterior probability

Phylogenetic analysis based on ITS sequences using MP and BI yielded similar tree topology. The tested nine endophytic fungal strains with the known *Muscodor* species formed a strong supported cluster with 90% MPBP and 1.00 BIPP (Figure 1). Y‐L‐56 clustered with *M. vitigenus*,* M. sutura*, and *M. equiseti* with 96% MPBP and 1.00 BIPP. N‐L‐7, Y‐L‐43, and W‐S‐35 clustered with *M. coffeanum* with 93% MPBP and 0.99 BIPP. W‐S‐38 formed an independent branch. W‐T‐27, Y‐S‐35, and *M. suthepensis* formed a clade with 100% MPBP and 1.00 BIPP. Y‐L‐54, W‐S‐41 and *M. albus*,* M. camphorae*,* M. oryzae*,* M. roseus*,* M. musae*,* M. ghoomensis*,* M. kashayum*,* M. tigerii*,* M. cinnamomi*,* M. darjeelingensis*,* M. strobelii*,* M. crispans*, and *M. indicus* clustered into a clade with 64% MPBP and 1.00 BIPP. Therefore, the nine tested endophytic fungal strains were identified as five different *Muscodor* species: *Muscodor* sp. 1 to *Muscodor* sp. 5 (Table 1). ITS sequence analysis also showed that *Muscodor* sp. 3 (W‐S‐38), *Muscodor* sp. 4 (N‐L‐7, Y‐L‐43, and W‐S‐35), and *Muscodor* sp. 5 (Y‐L‐56) have considerable sequence differences from known *Muscodor* species.

### 3.2 Characteristics of the tested *Muscodor* colonies and the optimal growth temperature

**Figure 2:**
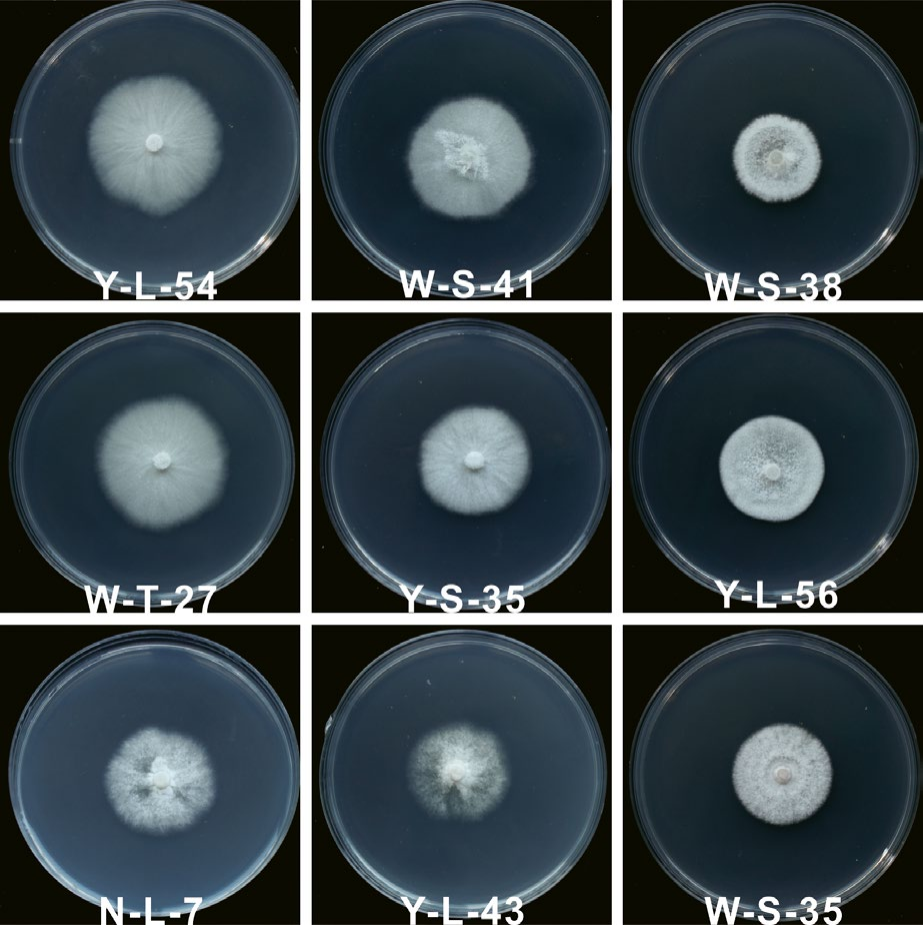
Colony of the tested *Muscodor* endophytes incubated at 25°C on potato dextrose agar for 15 days

Colonies of the tested *Muscodor* strains cultivated at 25°C on PDA medium are shown in Figure 2. All colonies were white, but the colony appearances of different strains were slightly different. Colonies of strains Y‐L‐54, W‐S‐41, W‐T‐27, and Y‐S‐35 were smooth with sparse aerial mycelia and radialized vegetative mycelia, while aerial mycelia of strains W‐S‐38, Y‐L‐56, and W‐S‐35 were floccus. Strains N‐L‐7 and Y‐L‐43 were wooly. None of the strains produced spores on PDA.

The growth temperature of the tested *Muscodor* strains cultivated on PDA medium is shown in Table 2. There was little difference among the optimal growth temperatures of the tested *Muscodor* strains on PDA. None of the tested *Muscodor* strains can grow at temperatures above 30°C. Seven of the *Muscodor* strains can still grow at 30°C, while the other two strains, W‐S‐38 and W‐S‐35, stopped growing. The optimal growth temperature range for the tested *Muscodor* strains is 20–28°C, except for W‐S‐38, which shows optimal growth at 20–25°C.

**Table 2:** The growth temperature of the *Muscodor* endophytes

Strain	Colony diameter (cm)					
	15°C	20°C	23°C	25°C	28°C	30°C
Y‐L‐54	1.8 ± 0.2	4.1 ± 0.1	4.7 ± 0.2	5.0 ± 0.5	4.2 ± 0.5	1.8 ± 0.2
W‐S‐41	1.7 ± 0.2	4.0 ± 0.3	4.1 ± 0.1	4.2 ± 0.2	3.4 ± 0.3	0.5 ± 0.2
W‐T‐27	1.6 ± 0.1	4.7 ± 0.1	5.7 ± 0.3	6.7 ± 0.6	5.1 ± 0.1	1.6 ± 0.3
Y‐S‐35	1.9 ± 0.1	3.4 ± 0.1	4.0 ± 0.1	4.1 ± 0.2	3.7 ± 0.3	1.8 ± 0.1
W‐S‐38	1.2 ± 0.1	2.9 ± 0.2	3.6 ± 0.1	4.6 ± 0.2	1.2 ± 0.1	0.0 ± 0.0
N‐L‐7	1.1 ± 0.0	3.1 ± 0.1	4.0 ± 0.2	4.5 ± 0.2	3.1 ± 0.2	1.1 ± 0.1
Y‐L‐43	1.1 ± 0.2	3.2 ± 0.1	4.1 ± 0.1	4.3 ± 0.2	3.6 ± 0.1	0.8 ± 0.1
W‐S‐35	1.1 ± 0.1	3.1 ± 0.1	3.8 ± 0.3	4.0 ± 0.1	3.4 ± 0.1	0.0 ± 0.0
Y‐L‐56	1.0 ± 0.2	3.2 ± 0.2	3.6 ± 0.3	3.6 ± 0.1	3.9 ± 0.2	1.2 ± 0.2

### 3.3 Antifungal activity of the tested *Muscodor* strains

The antifungal activity of the tested *Muscodor* strains was shown in Table 3. The VOCs of *Muscodor* strain W‐S‐41 showed the strongest antifungal activity, with complete inhibition and killing of mycelial growth of *Botrytis cinerea*,* Penicillium digitatum*, and *Pythium ultimum*. In addition, the VOCs of *Muscodor* strain W‐S‐41 was able to effectively inhibit two other pathogens: *Fusarium oxysporum* and *Pestalotia diospyr*. The VOCs of *Muscodor* strain N‐L‐7 showed the weakest antifungal activity, only partially or slightly inhibiting *Penicillium digitatum*,* Botrytis cinerea*, and *Pestalotia diospyr*, and showing no inhibition activity on *Fusarium oxysporum* and *Pythium ultimum*.

**Table 3:** Effects of the volatile compounds of *Muscodor* strains on the tested plant pathogenic fungi

Group	Strain	*Botrytis cinerea*		*Fusarium oxysporum*		*Penicillium digitatum*		*Pestalotia diospyr*		*Pythium ultimum*	
		Inhibitory rate, %	Viability	Inhibitory rate, %	Viability	Inhibitory rate, %	Viability	Inhibitory rate, %	Viability	Inhibitory rate, %	Viability
I	W‐S‐41	100.00	Dead	76.77	Alive	100.00	Dead	44.44	Alive	100.00	Dead
	Y‐L‐54	100.00	Dead	59.60	Alive	100.00	Dead	41.03	Alive	100.00	Dead
	Y‐S‐35	100.00	Dead	53.54	Alive	100.00	Dead	39.74	Alive	100.00	Dead
	W‐T‐27	100.00	Dead	50.00	Alive	100.00	Dead	38.03	Alive	100.00	Dead
	Y‐L‐56	100.00	Alive	37.37	Alive	100.00	Dead	22.65	Alive	100.00	Dead
II	W‐S‐35	66.48	Alive	23.23	Alive	100.00	Alive	12.82	Alive	100.00	Alive
III	Y‐L‐43	74.41	Alive	0	Alive	4.56	Alive	2.56	Alive	100.00	Alive
IV	W‐S‐38	29.31	Alive	37.37	Alive	23.47	Alive	31.20	Alive	0	Alive
	N‐L‐7	17.12	Alive	0	Alive	41.22	Alive	14.96	Alive	0	Alive

Among the targeted pathogens, *P. ultimum* was the most sensitive to VOCs produced by *Muscodor*. When exposed to the VOCs produced by *Muscodor*,* P. ultimum* was completely inhibited and even killed by seven of the VOCs. *Penicillium digitatum* (sensitive to six VOCs) and *B. cinerea* (sensitive to five VOCs) also showed sensitivity to the VOCs produced by *Muscodor*. *Fusarium oxysporum* and *P. diospyr* were the least sensitive to the VOCs of *Muscodor*; they were only partially inhibited by the VOCs.

Through the SPSS K‐means cluster analysis based on antifungal activities, the tested *Muscodor* strains can be divided into four groups: I–IV. The first group contains the strains W‐S‐41, Y‐L‐54, Y‐S‐35, W‐T‐27, and Y‐L‐56, which showed the strongest antifungal activity. These strains completely inhibited and killed *B. cinerea*,* P. digitatum*, and *P. ultimum* and moderately or partially inhibited *F. oxysporum* and *P. diospyr*. Strain W‐S‐35 belongs to the second group, which showed stronger antifungal activity. It completely inhibited *P. digitatum* and *P. ultimum*, moderately inhibited *B. cinerea*, and partially inhibited *F. oxysporum* and *P. diospyr*. The third group contains strain Y‐L‐43, which showed weak antifungal activity. It completely inhibited *P. ultimum*, moderately inhibited *B. cinerea*, and weakly or did not inhibit *F. oxysporum*,* P. digitatum*, and *P. diospyr*. The fourth group, contains strains W‐S‐38 and N‐L‐7, which were the weakest at inhibiting the five tested pathogens.

### 3.4 The components and the relative amounts of VOCs produced by the tested *Muscodor* strains

Thirty‐five compounds and the relative amounts of VOCs were determined (Table 4), which were classified into two categories. One is the product of fatty acid degradation, such as methyl isobutyrate, 3‐methyl‐1‐butanol, 2‐methylpropanoic acid, 3‐methyl‐1‐butyl acetate, 1‐octen‐3‐ol, 3‐octanone, and 2‐nonanone. The other category is terpenes, such as monoterpenes (1‐ethenyl‐1‐methyl‐2,4‐bis(1‐methylethenyl)‐cyclohexane) and sesquiterpenes (caryophyllene), which are the intermediate and final metabolites of the metabolic pathway with the precursor of mevalonic acid.

**Table 4:** The volatile organic compounds produced by *Muscodor* strains through SPME/GC/MS analysis

No.	RT (min)	Possible compound^a^	CAS no.	NIST no.	Lib. match score	*Mr*	Peak area^b^								
							W‐S‐41	Y‐L‐54	Y‐S‐35	W‐T‐27	Y‐L‐56	W‐S‐35	Y‐L‐43	W‐S‐38	N‐L‐7
com1	3.448	Propanoic acid, 2‐methyl‐, methyl ester	547‐63‐7	34178	920	102	612879	446802	925157	472660	144209	290250	/	/	/
com2	4.799	1‐Butanol, 3‐methyl‐	123‐51‐3	19490	843	88	/	/	/	/	/	/	/	809102	/
com3	8.148	Propanoic acid, 2‐methyl‐	79‐31‐2	19501	917	88	708184	4003722	8386293	4672700	1930881	2476244	/	/	/
com4	9.521	1‐Butanol, 3‐methyl‐, acetate	123‐92‐2	291294	935	130	113689	66375	48349	/	/	/	/	81334	/
com5	12.983	1‐Octen‐3‐ol	3391‐86‐4	19422	890	128	/	/	/	/	/	306613	/	/	/
com6	13.207	3‐Octanone	106‐68‐3	163610	912	128	/	/	/	/	/	170551	/	/	/
com7	16.001	4‐Nonanone	4485‐09‐0	114360	926	142	/	21555	16037	46809	/	/	/	58463	/
com8	16.612	2‐Nonanone	821‐55‐6	114362	830	142	/	18828	/	21940	/	/	/	/	/
com9	16.690	Propanoic acid, 2‐methyl‐, pentyl ester	2445‐72‐9	280486	780	158	/	/	17140	17707	/	/	/	/	/
com10	17.257	Octen‐1‐ol, acetate	32717‐31‐0	6634	929	170	/	/	/	/	/	157307	/	/	/
com11	18.755	Octanoic acid, 2‐methyl‐, methyl ester	2177‐86‐8	62444	821	172	/	/	/	/	/	/	/	22396	/
com12	21.148	Bicyclo[2.2.1]heptane, 2‐methylene‐3‐(1‐methylethenyl)‐	77764‐41‐1	150031	764	148	/	/	/	/	17343	/	/	/	/
com13	21.498	Acetic acid, 2‐phenylethyl ester	103‐45‐7	107577	894	164	36042	49418	89273	22125	/	24542	/	32815	/
com14	21.765	Tridecane, 7‐methylene‐	19780‐80‐4	113992	795	196	/	/	/	/	/	/	/	21168	/
com15	23.252	1H‐Cyclopropa[a]naphthalene, 1a,2,3,3a,4,5,6,7b‐octahydro‐1,1,3a,7‐tetramethyl‐, [1aR‐(1aà,3aà,7bà)]‐	489‐29‐2	9244	847	204	/	/	/	/	/	/	/	/	10757
com16	24.136	Caryophyllene‐(I1)	N/A	158185	824	204	/	/	/	/	/	/	12852	/	31956
com17	24.456	Biphenylene, 1,2,3,6,7,8,8a,8b‐octahydro‐4,5‐dimethyl‐	106988‐87‐8	142222	819	188	/	/	/	/	/	/	13314	/	/
com18	24.899	1H‐Cyclopropa[a]naphthalene, 1a,2,3,5,6,7,7a,7b‐octahydro‐1,1,7,7a‐tetramethyl‐ [1aR‐(1aà,7à,7aà,7bà)]‐	17334‐55‐3	249534	845	204	/	/	/	/	/	14535	33139	/	70109
com19	25.016	(‐)‐Tricyclo[6.2.1.0(4,11)]undec‐5‐ene, 1,5,9,9‐tetramethyl‐ (isocaryophyllene‐I1)	N/A	154067	847	204	/	/	/	/	/	/	59052	/	29412
com20	25.208	Cyclohexane, 1‐ethenyl‐1‐methyl‐2,4‐bis(1‐methylethenyl)‐, [1S‐(1à,2á,4á)]‐	515‐13‐9	22550	928	204	/	/	/	/	/	/	/	26202	/
com21	25.466	4,4‐Dimethyl‐3‐(3‐methylbut‐3‐enylidene)‐2‐methylenebicyclo[4.1.0]heptane	79718‐83‐5	195379	755	202	/	/	/	/	/	/	13282	/	21468
com22	25.763	1H‐3a,7‐Methanoazulene, 2,3,4,7,8,8a‐hexahydro‐3,6,8,8‐tetramethyl‐ [3R‐(3à,3aá,7á,8aà)]‐	469‐61‐4	22526	924	204	13487	/	/	30534	/	/	/	/	/
com23	26.307	Bicyclo[3.1.1]hept‐2‐ene, 2,6‐dimethyl‐6‐(4‐methyl‐3‐pentenyl)‐	17699‐05‐7	141044	935	204	10461	26174	27232	11430	/	19950	/	/	24561
com24	26.392	Azulene, 1,2,3,4,5,6,7,8‐octahydro‐1,4‐dimethyl‐7‐(1‐methylethenyl)‐, [1S‐(1à,4à,7à)]‐	3691‐12‐1	9225	936	204	16491	/	10527	/	/	/	/	14387	/
com25	27.461	Bicyclo[7.2.0]undec‐4‐ene, 4,11,11‐trimethyl‐8‐methylene‐[1R‐(1R*,4Z,9S*)]‐	118‐65‐0	249403	885	204	/	13040	/	/	/	76483	/	/	/
com26	27.551	1,6,10‐Dodecatriene, 7,11‐dimethyl‐3‐methylene‐ (Z)‐	28973‐97‐9	141110	901	204	/	/	/	/	/	/	36032	/	129446
com27	27.677	2‐Isopropenyl‐4a,8‐dimethyl‐1,2,3,4,4a,5,6,8a‐octahydronaphthalene	N/A	193570	911	204	49505	/	10706	/	/	/	/	40370	/
com28	27.936	1R,3Z,9S‐2,6,10,10‐Tetramethylbicyclo[7.2.0]undeca‐2,6‐diene	N/A	140074	851	204	24180	/	/	/	/	/	/	20364	/
com29	27.987	Calarene epoxide	N/A	151460	745	220	/	/	/	/	/	16160	38668	/	72480
com30	28.083	Azulene, 1,2,3,5,6,7,8,8a‐octahydro‐1,4‐dimethyl‐7‐(1‐methylethenyl)‐ [1S‐(1à,7à,8aá)]‐	3691‐11‐0	70226	948	204	251410	30941	36361	17437	/	/	/	211194	10861
com31	28.648	Spiro[5.5]undeca‐1,8‐diene, 1,5,5,9‐tetramethyl‐ (R)‐	19912‐83‐5	249575	814	204	/	/	/	/	/	90834	118980	/	321594
com32	29.427	Patchouli alcohol	5986‐55‐0	141042	796	222	/	/	/	/	/	/	/	18101	/
com33	31.561	1H‐Cycloprop[e]azulen‐4‐ol, decahydro‐1,1,4,7‐tetramethyl‐ [1ar‐(1aà,4á,4aá,7à,7aá,7bà)]‐	552‐02‐3	141116	857	222	342257	175943	344106	129530	/	13024	/	120395	/
com34	34.293	2‐Propenoic acid, 3‐(4‐methoxyphenyl)‐, 2‐ethylhexyl ester	5466‐77‐3	291525	921	290	/	/	/	/	/	/	/	793815	/
com35	37.096	Hexadecanoic acid, methyl ester	112‐39‐0	158970	886	270	/	11735	14460	/	/	17901	/	/	/

a The chemical nomenclature was adopted according to NIST14 database.

b The corresponding compounds existed in controls were deleted. Minor peaks with signal‐to‐noise ratio <3 were cutoff.

**Figure 3:**
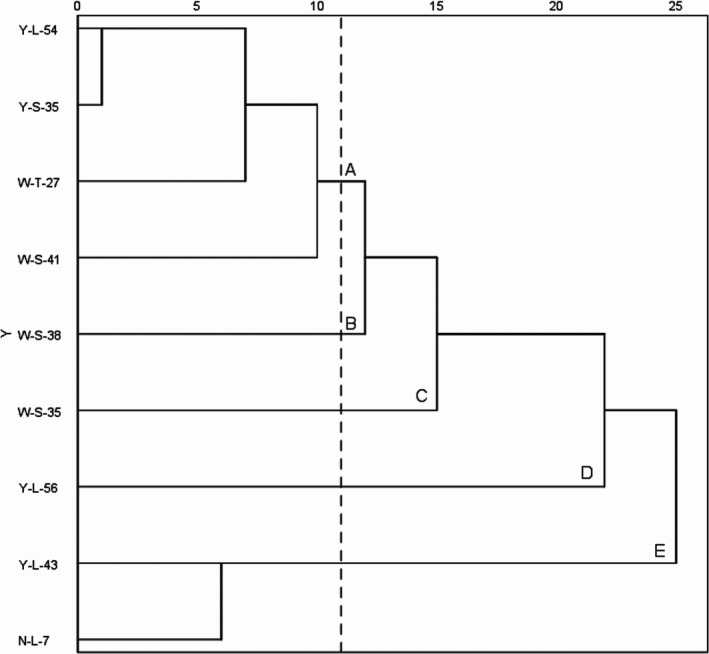
The chemical dendrogram of *Muscodor* strains. The vertical axis represents the strains and the horizontal axis represents the distance index. Five clusters were divided with a dashed line between W‐S‐41 and W‐S‐38. Both cluster A (the strains Y‐L‐54, Y‐S‐35, W‐T‐27, and W‐S‐41) and the cluster D (strain Y‐L‐56) showed the strongest antifungal activity. Cluster C (the strain W‐S‐35) showed stronger antifungal activity. The cluster B (strain W‐S‐38) showed the weakest antifungal activity. The cluster E (strains N‐L‐7 and Y‐L‐43) showed the weakest antifungal activity

A chemical dendrogram (Figure 3) was created using SPSS clustering analysis. The dendrogram showed that components of the VOCs from Y‐L‐54 and Y‐S‐35 were similar. In addition, the components of the VOCs from Y‐L‐43 and N‐L‐7 were also similar.

## 4 DISCUSSION

Typical fungal identification mainly depends on morphological characters and phylogenetic analyses. The identification of *Muscodor* can be challenging, as *Muscodor* spp. do not produce sporogenous structures. Therefore, phylogentic analyses are essential tool for identification of *Muscodor*. Owing to the lack of informative sequences of the reference species except for ITS, the ITS sequence analysis in this study was carried out and was able to identify the tested strains as *Muscodor*, and support the strains as belonging to five different *Muscodor* species. Nonetheless, ITS is not diverged enough to informatively discern the phylogenetic relationships among *Muscodor* species. In a study on xylariaceous fungi, Jaklitsch & Voglmayr interpreted the phylogenetic relationships of five genera from Xylariales, employing ITS, LSU, rpb2, and tef1 (Jaklitsch & Voglmayr, 2012). Thus, further multigene phylogenetic analyses are required for interpretation of the phylogenetic relationships among *Muscodor* species and designation species names for these strains.

**Figure 4:**
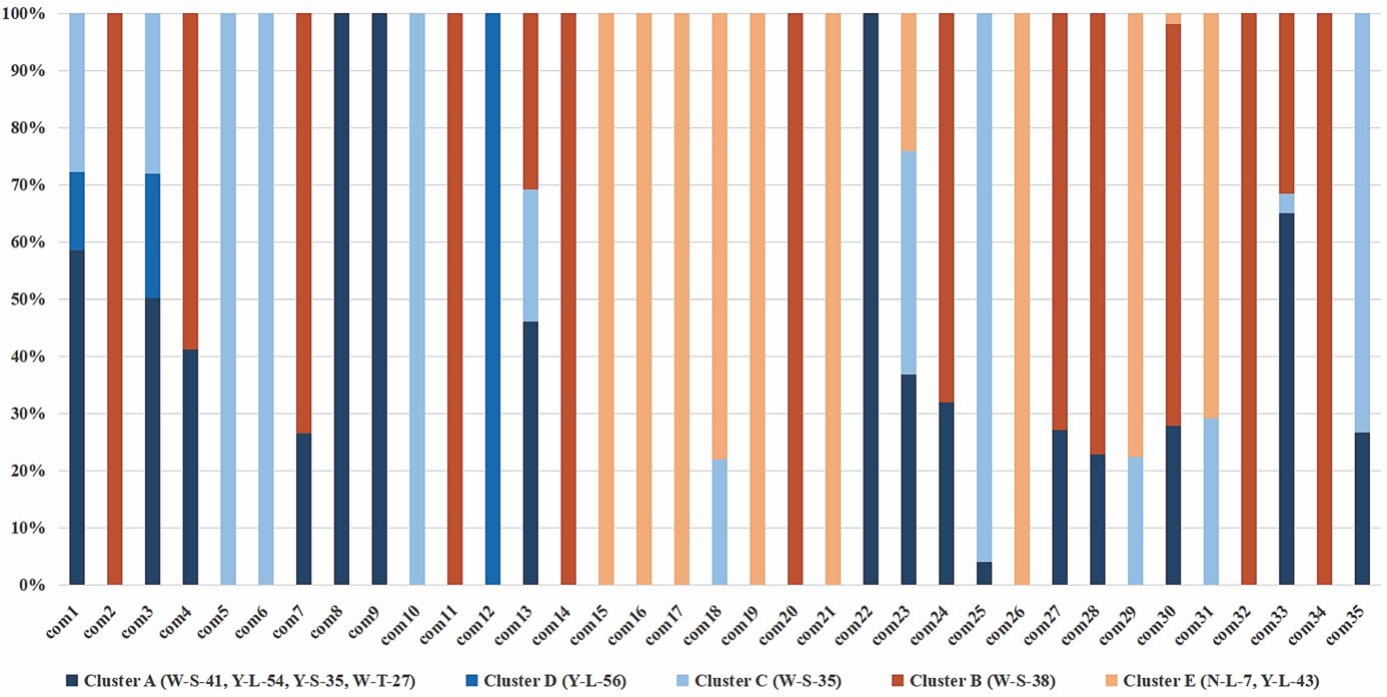
The peak area percent of volatile organic compound components of the *Muscodor* clusters. The vertical axis represents the percentage of the compound in clusters and the horizontal axis represents the compound. Cluster A (strains W‐S‐41, Y‐L‐54, Y‐S‐35, and W‐T‐27) and cluster D (strain Y‐L‐56), which showed the strongest antifungal activity, are marked with dark blue and blue, respectively. Cluster C (strain W‐S‐35), which showed stronger antifungal activity, is marked with light blue. Cluster B (strain W‐S‐38), which showed the weakest antifungal activity, is marked with orange. Cluster E (strains N‐L‐7 and Y‐L‐43), which showed weak or the weakest antifungal activity, is marked with yellow

We found that the Y‐L‐54 and Y‐S‐35 strains, which belong to different *Muscodor* species, produce the most similar VOC components. On the other hand, N‐L‐7, Y‐L‐43, and W‐S‐35, which are strains of the same *Muscodor* species, produce different VOC components. This indicates that the VOCs might not be correlated with their phylogenetic assignment, at least among these *Muscodor* strains. Thus, the five clusters were divided with a dashed line according to antifungal activity (Figure 3). Cluster A (strains Y‐L‐54, Y‐S‐35, W‐T‐27, and W‐S‐41) and cluster D (strain Y‐L‐56), which showed the strongest antifungal activity. Cluster C (strain W‐S‐35), which showed stronger antifungal activity. Cluster B (strain W‐S‐38) and Cluster E (strains N‐L‐7 and Y‐L‐43) showed weak or the weakest antifungal activity. The peak area percentage of VOC components for the five clusters was obtained (Figure 4), and the specific compounds (com1, com3, com5, com6, com8, com 9, com 10, com 12, com22, com25, and com35) in the clusters marked with varying shades of blue might be responsible for antifungal activity, and these compounds are mostly esters, alcohols, and small molecular weight acids, in particular, methyl isobutyrate and 2‐methylpropanoic acid, which were produced by all highly active strains (strains W‐S‐41, Y‐L‐54, Y‐S‐35, W‐T‐27, Y‐L‐56, and W‐S‐35). The most active group of compounds identified in this study are consistent with the previous study that the esters, alcohols, and acids produced by *Muscodor* spp. are remarkable bioactive properties (Mitchel et al., 2008; Strobel et al., 2001).

This study found that *Muscodor* endophytes are widespread in tropical gramineous plants and a number of strains can inhibit or kill a variety of plant pathogens. Therefore, they have important potential applications in the fields of plant disease control. Moreover, endophytic *Muscodor* species interacting with host plants, possibly leads to the change in VOCs of the host plants. Different VOCs have different biological effects; for example, methyl isobutyrate and 2‐methylpropanoic acid have stronger antifungal activity. On the other hand, caryophyllene has been shown to attract insects (Xiao et al., 2012). Thus, the ecological effects of *Muscodor* endophytes and plant interactions deserve further study.

## CONFLICT OF INTEREST

The authors state that there are no conflicts of interest.
